# An optogenetic tool to recruit individual PKC isozymes to the cell surface and promote specific phosphorylation of membrane proteins

**DOI:** 10.1016/j.jbc.2022.101893

**Published:** 2022-03-31

**Authors:** Kirin D. Gada, Takeharu Kawano, Leigh D. Plant, Diomedes E. Logothetis

**Affiliations:** 1 Department of Pharmaceutical Sciences, Bouvé College of Health Sciences and College of Science, Northeastern University, Boston, Massachusetts, USA; 2 Center for Drug Discovery, Bouvé College of Health Sciences and College of Science, Northeastern University, Boston, Massachusetts, USA; 3 Department of Chemistry and Chemical Biology, Bouvé College of Health Sciences and College of Science, Northeastern University, Boston, Massachusetts, USA

**Keywords:** PKC, optogenetics, ion channel, phosphorylation, membrane protein, CAT, catalytic domain, cPKC, conventional PKC, CIB1, calcium and integrin-binding protein 1, CIBN, N-terminal region of the CRY2-binding domain of CIB1, CRY2, cryptochrome-2, DAG, diacylglycerol, GIRK1/4, G protein–gated inwardly rectifying K^+^ channels 1 and 4, HA, hemagglutinin, HEK-293T, human embryonic kidney 293T cell, nPKC, novel PKC, PEI, polyethyleneimine, TIRFM, total internal reflection fluorescence microscopy

## Abstract

The PKC family consists of several closely related kinases. These enzymes regulate the function of proteins through the phosphorylation of hydroxyl groups on serines and/or threonines. The selective activation of individual PKC isozymes has proven challenging because of a lack of specific activator molecules. Here, we developed an optogenetic blue light–activated PKC isozyme that harnesses a plant-based dimerization system between the photosensitive cryptochrome-2 (CRY2) and the N terminus of the transcription factor calcium and integrin-binding protein 1 (CIB1) (N-terminal region of the CRY2-binding domain of CIB1). We show that tagging CRY2 with the catalytic domain of PKC isozymes can efficiently promote its translocation to the cell surface upon blue light exposure. We demonstrate this system using PKCε and show that this leads to robust activation of a K^+^ channel (G protein–gated inwardly rectifying K^+^ channels 1 and 4), previously shown to be activated by PKCε. We anticipate that this approach can be utilized for other PKC isoforms to provide a reliable and direct stimulus for targeted membrane protein phosphorylation by the relevant PKCs.

The PKC enzyme family consists of serine–threonine kinases that are activated downstream of phosphoinositide hydrolysis (1). PKC was first isolated in rodent and bovine brain fractions, almost 5 decades after phosphoserine was first discovered by Fritz Lipmann (2). Initially named PKM because of its perceived dependence on Mg^2+^, this enzyme was renamed PKC after its regulation by Ca^2+^ was discovered.

PKC isoforms share the same basic structure with a conserved catalytic domain at their C terminus (PKC-CAT) and a membrane-targeting segment at the N-terminal end of the protein (Fig. 1*A*). The enzyme sequence is further divided into C1, C2, C3, and C4 from the N- to C-terminal ends. C1 and C2 are part of the regulatory domain of the enzyme, with C1 (C1A and C1B) being the binding region for phorbol esters and anionic lipids and C2 serving as a Ca^2+^ sensor. The conserved catalytic domain consists of an ATP-binding region, C3, and a substrate-binding region, C4. C3 and C4 are separated from the regulatory domain by a flexible hinge that is susceptible to proteolytic cleavage to yield a constitutively active enzyme (3). All the isozymes harbor a pseudosubstrate sequence that subdues the enzyme in its inactive state by engaging the substrate-binding cavity (1).

**Figure 1:**
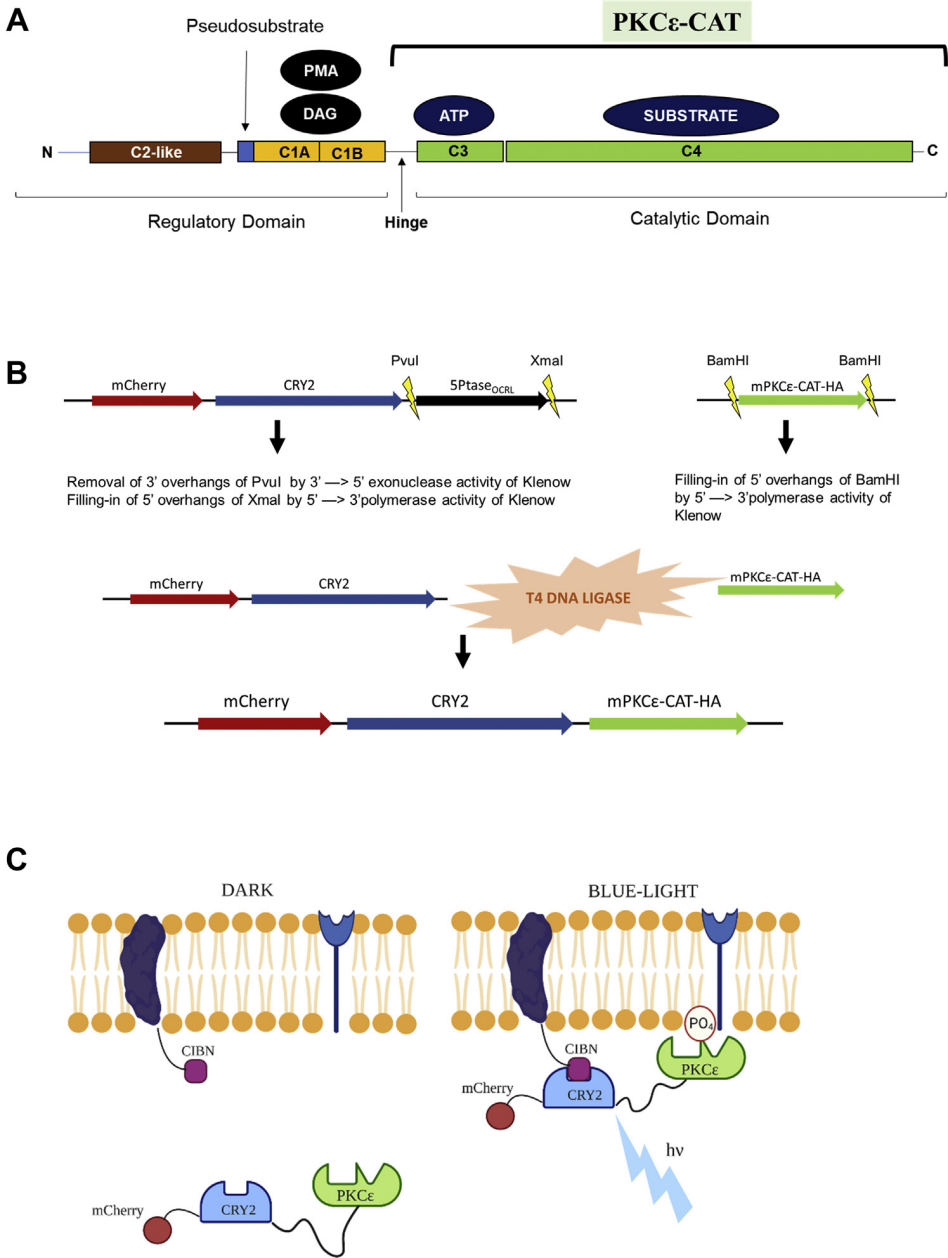
**Schematic drawing depicting constructs involved in optogenetic activation of PKCε-CAT.***A*, schematic of the subdomains within the PKCε isozyme. *B*, digestion of parent constructs mCherry-CRY2-5Ptase_OCRL_ and mPKCε-CAT-HA-pXOOM; ligation of fragments with T4 DNA ligase. *C*, *blue light* induced recruitment of mCherry-CRY2–mPKCε-CAT-HA to the cell surface and the subsequent phosphorylation of membrane protein substrates by PKCε. CAT, catalytic domain; CRY2, cryptochrome-2; HA, hemagglutinin.

Based on their domain composition, the PKC enzyme family is divided into three different subtypes, conventional PKCs (cPKCs), novel PKCs (nPKCs), and atypical PKCs, which are encoded in nine genes (2). Contingent on the cofactors needed for activation and the anatomy of their regulatory domain, PKC isoforms α, βI, βII, and γ are classified under cPKC (4). These enzymes are known as Ca^2+^-sensitive PKCs because of the presence of acidic amino acids in their C2 domain that bind Ca^2+^. cPKCs are targeted to the membrane after Ca^2+^ binding (4). cPKCs possess a binding surface for phosphatidylinositol 4,5 bisphosphate, a lipid only found in the inner plasma membrane, on their C2 domain, the binding of which is integral to the release of the pseudosubstrate to initiate kinase activity.

nPKCs include ε, δ, θ, and η and have a twofold higher affinity for diacylglycerol (DAG) than cPKCs. These isozymes lack the acidic amino acids in the C2-like novel C2 domain and are not dependent on Ca^2+^ for activity (shown in Fig. 1*A* for PKCε). nPKCs have the highest affinity of all PKCs for DAG and are primarily recruited to the DAG-rich Golgi membrane (4). PKCs ζ and ι are classified as atypical PKCs since they are Ca^2+^ insensitive as well as independent of DAG.

Several compounds like phorbol esters, bryostatin, and DAG activate PKCs. Phorbol esters are well-known PKC activators; however, the cell’s inability to metabolize these compounds results in the sustained activation of PKC, which eventually causes enzyme downregulation (5). Other activators of PKCs are available; however, they do not all exclusively activate PKCs and are reported to have some off-target effects on other enzymes (6). Another drawback of small-molecule activators is the inability to target the enzyme to specific cellular compartments. While cPKCs are recruited to the cell membrane, nPKCs are primarily recruited to DAG-rich membranes, like the Golgi apparatus, because of their extremely high affinity for DAG (4). This presents a significant limiting factor for PKC isozyme-specific studies since nPKCs have been reported to phosphorylate membrane proteins like ion channels (*e.g.*, G protein–gated inwardly rectifying K^+^ channels 1 and 4 [GIRK1/4]) (7).

The development of optogenetic tools has been attempted in the past to enable dynamic monitoring of PKC translocation to the cell surface and phosphorylation of membrane proteins, such as ion channels (8); however, they have not been utilized extensively. We have employed a blue light–activated optogenetic tool used to acutely manipulate membrane phosphatidylinositol phosphates (5) and have found it to translocate 5-Ptase_OCRL_ to the plasma membrane rapidly and with a high degree of spatiotemporal precision (9). Hence, we developed a similar tool based on this system to target PKC enzymes to the plasma membrane to study their effects on membrane proteins. This optogenetic platform takes advantage of a light-controlled dimerization system based on two plant proteins, the photosensitive cryptochrome-2 (CRY2) and the transcription factor calcium and integrin-binding protein 1 (CIB1). Blue light illumination causes the absorption of an FAD molecule and a consequent conformational change in the CRY2 photolyase homology region that promotes its binding to the N-terminal region of the CRY2-binding domain of CIB1 (CIBN). The interaction of these proteins regulates a subset of genes in plants that are responsible for floral initiation, first described by Liu *et al.* (10). The system developed by Idevall-Hagren *et al.* (5) is comprised of two fusion proteins, mCherry-CRY2-5-Ptase_OCRL_ and CIBN–CAAX. mCherry-CRY2-5-Ptase_OCRL_ contains the photolyase domain of CRY2 and the inositol 5-phosphatase domain of the Lowe oculocerebrorenal syndrome protein (OCRL). CIBN–CAAX contains the CIBN and a C-terminal CAAX box for plasma membrane targeting by the addition of a lipid to the Cys residue in CAAX. Dimerization between CRY2 and CIBN results in the translocation of the CRY2-linked enzyme to the plasma membrane. Here, we illustrate the utility of this strategy for PKC isozymes through the development of the optogenetic recruitment of the catalytic domain of the nPKC isozyme, PKCε, to the plasma membrane, and its consequent regulation of the cardiac GIRK1/4.

## Results

The nPKC, PKCε, has a regulatory domain and a catalytic domain separated by a hinge region (Fig. 1*A*). The catalytic domain of PKCε—mPKCε-CAT-hemagglutinin (HA)—was fused to CRY2 (Fig. 1*B*) and mCherry to generate mCherry-CRY2–mPKCε-CAT-HA. The development of this construct is detailed in the Experimental procedures section. Figure 1*C* depicts the mechanism of action of the engineered optogenetic system. Upon illumination with blue light (∼445 nm), the CRY2-associated PKCε translocates to the cell surface and undertakes phosphorylation of membrane protein substrates.

### mCherry-CRY2–mPKCε-CAT-HA translocates to the plasma membrane

**Figure 2:**
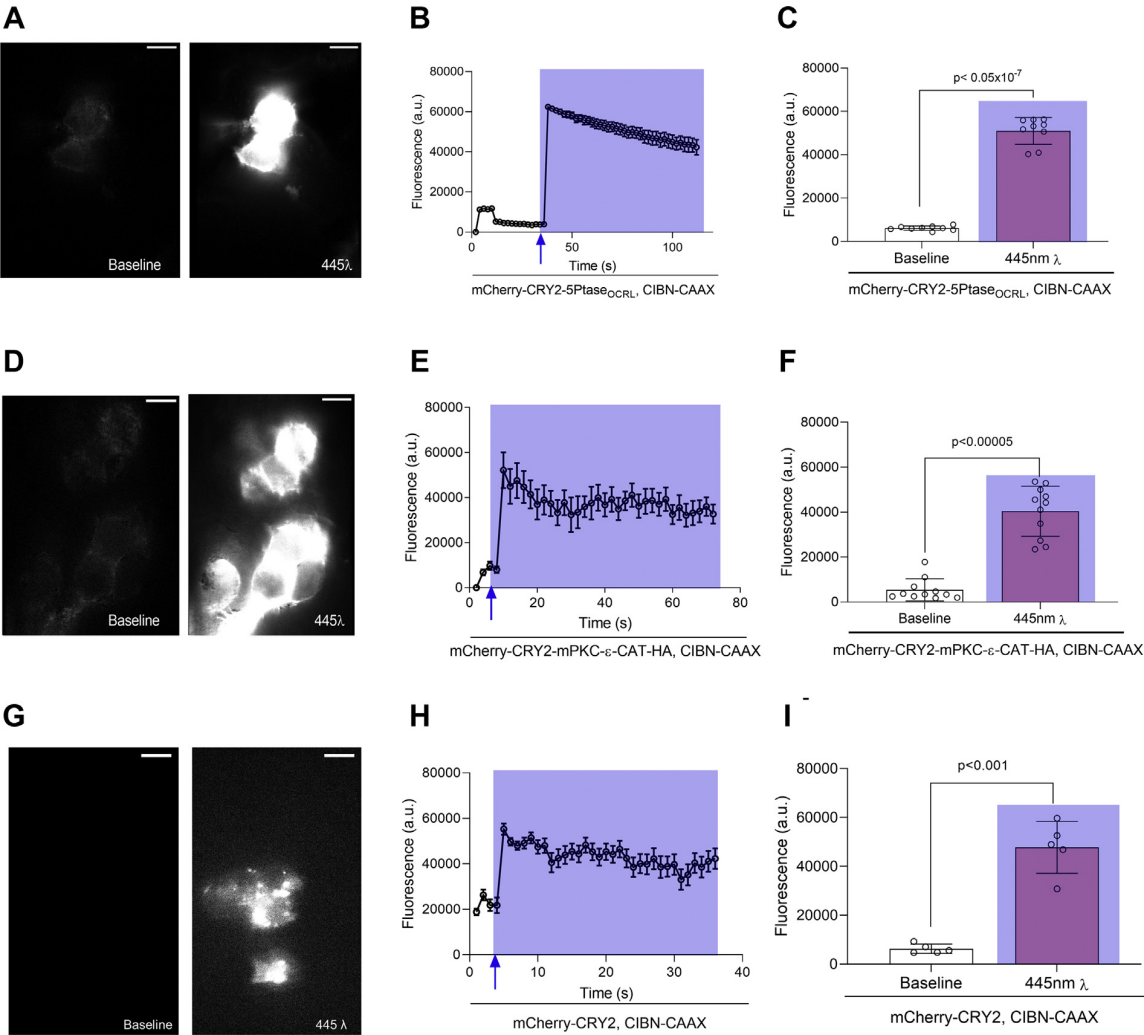
**mCherry-CRY2–mPKCε-CAT-HA translocates to the cell membrane when exposed to blue light.** About 0.75 μg of mCherry-CRY2-5Ptase_OCRL_ was coexpressed with 0.75 μg CIBN–CAAX in HEK-293T cells. *A*, representative TIRFM images showing the surface fluorescence of cells transfected with mCherry-CRY2-5Ptase_OCRL_ and CIBN–CAAX before and after illumination with a *blue* laser. *B*, a time course of cell surface fluorescence shows an increase in mCherry fluorescence at the cell membrane. *C*, summary data showing the surface fluorescence of cells before and after illumination with a blue laser. About 0.75 μg of mCherry-CRY2–mPKCε-CAT-HA was coexpressed with 0.75 μg CIBN–CAAX in HEK-293T cells. *D*, representative TIRFM images showing the surface fluorescence of cells transfected with mCherry-CRY2–mPKCε-CAT-HA and CIBN–CAAX before and after illumination with a blue laser. *E*, a time course of cell surface fluorescence shows an increase in mCherry fluorescence at the cell membrane. *F*, summary data showing the surface fluorescence of cells before and after illumination with a *blue* laser. *G*, representative TIRFM images showing the surface fluorescence of cells transfected with mCherry-CRY2-5Ptase_OCRL_ and CIBN–CAAX before and after illumination with a *blue* laser. *H*, a time course of cell surface fluorescence shows an increase in mCherry fluorescence at the cell membrane. *I*, summary data showing the surface fluorescence of cells before and after illumination with a *blue* laser. Summary data are mean fluorescence ± SD for 5–11 cells per experiment; the scale bars represent 10 μM; and *p* Values are calculated using Student’s paired *t* test. CIBN, N-terminal region of the CRY2-binding domain of CIB1; CRY2, cryptochrome-2; HA, hemagglutinin; HEK-293T, human embryonic kidney 293T cell; TIRFM, total internal reflection fluorescence microscopy.

We used total internal reflection fluorescence microscopy (TIRFM) to image the cell surface and assess local recruitment of the mCherry-CRY2–mPKCε-CAT-HA to the cell membrane. The surface of cells transfected with mCherry-CRY2–mPKCε-CAT-HA and CIBN–CAAX (Fig. 2, *A*–*C*) or mCherry-CRY2-5Ptase_OCRL_ and CIBN–CAAX (Fig. 2, *D*–*F*) was brought into focus, after which a 561 nm laser (Coherent) was used to excite mCherry fluorescence. After the initiation of data acquisition, the cell was illuminated with a 445 nm laser (Coherent) to initiate the recruitment of the respective mCherry-CRY2–tagged fragments to the inner leaflet of the plasma membrane. Figure 2, *D*–*F* shows the recruitment of mCherry-CRY2–mPKCε-CAT-HA to the cell membrane following excitation by a “blue” laser. The translocation of mCherry-CRY2–mPKCε-CAT-HA to the cell membrane was comparable to mCherry-CRY2-5Ptase_OCRL_ (Fig. 2, *A*–*C*). The time course from the time of blue light illumination to change in the fluorescence intensity of mCherry, which is attached to CRY2-mPKCε-CAT-HA (Fig. 2*E*) and CRY2-5Ptase_OCRL_ (Fig. 2*B*), at the cell surface is within the 5 s sampling frequency we used for these studies. These data exemplify the rapid blue light–induced translocation of the engineered PKCε to the cell membrane.

The ability of the CRY2–CIBN system to localize to the cell membrane is exemplified in Figure 2 in the absence of an enzyme tagged to CRY2, showing that the addition of an enzyme does not meaningfully alter the efficiency of this optogenetic system.

### Neither CIBN–CAAX nor mCherry-CRY2–mPKCε-CAT-HA alone translocate to the plasma membrane comparably to the pair

**Figure 3:**
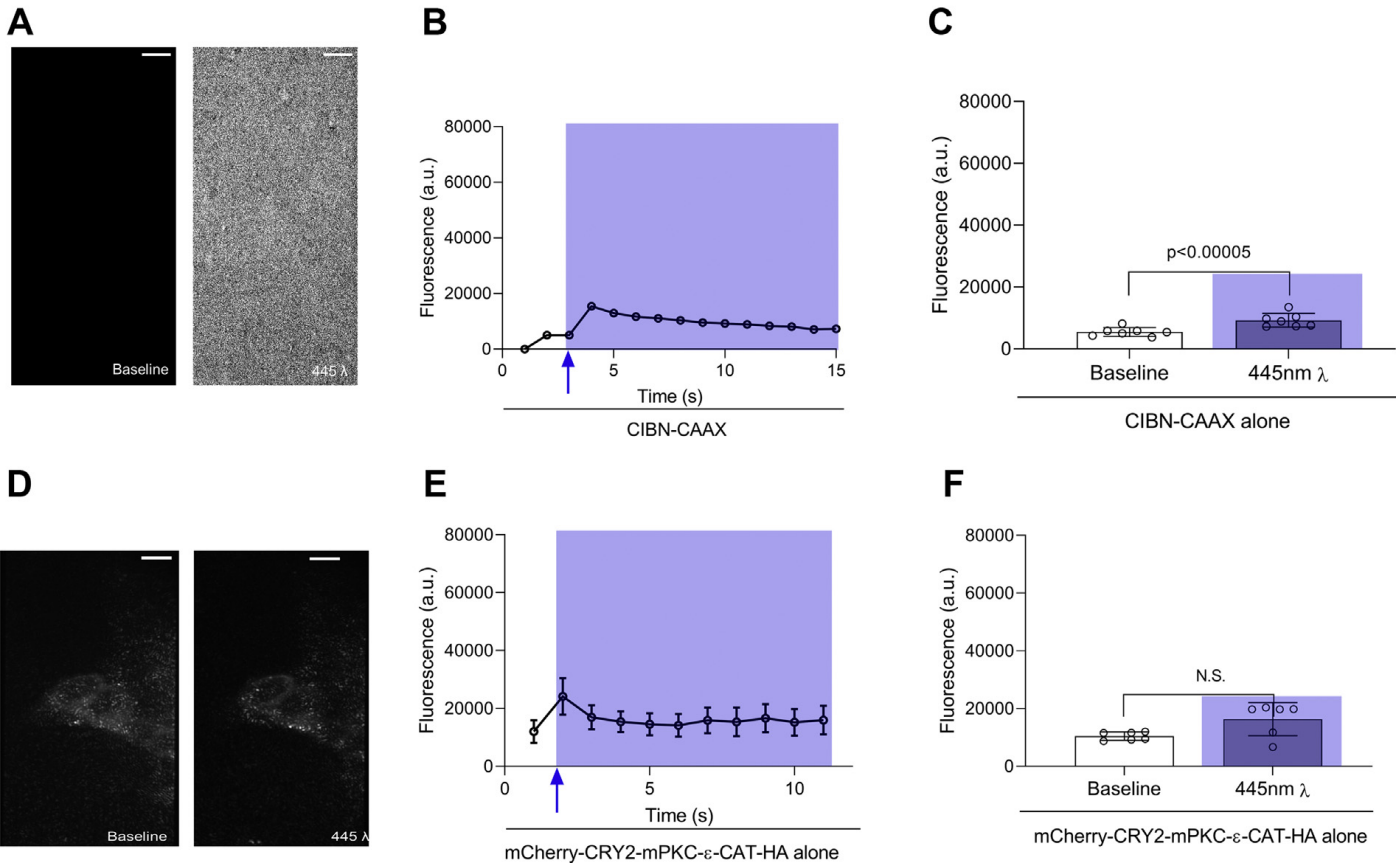
**Neither CIBN–CAAX nor mCherry-CRY2–mPKCε-CAT-HA is effective alone.** About 0.75 μg CIBN–CAAX was expressed in HEK-293T cells. Fluorescence was evaluated under excitation with a 561λ laser, before and after illumination with a *blue* laser (445λ). *A*, representative TIRFM images showing the surface fluorescence of cells transfected with CIBN–CAAX alone before and after illumination with a *blue* laser. *B*, a time course of cell surface fluorescence shows a small deviation from the baseline fluorescence, indicative of noise and backscatter of photons from the sample. *C*, summary data showing the surface fluorescence of cells before and after illumination with a *blue* laser. *D*, representative TIRFM images showing the surface fluorescence of cells transfected with mCherry-CRY2–mPKCε-CAT-HA alone before and after illumination with a *blue* laser. *E*, a time course of cell surface fluorescence. *F*, summary data showing the surface fluorescence of cells before and after illumination with a *blue* laser. Summary data are mean fluorescence ± SD for six to seven cells per experiment; scale bars represent 10 μM; and *p* values are calculated using Student’s paired *t* test. CAT, catalytic domain; CIBN, N-terminal region of the CRY2-binding domain of CIB1; CRY2, cryptochrome-2; HA, hemagglutinin; HEK-293T, human embryonic kidney 293T cell; TIRFM, total internal reflection fluorescence microscopy.

We can monitor recruitment of mCherry at the cell membrane a few seconds after illumination with the “blue” laser. We established a baseline for the TIRFM experiments to account for noise and backscatter of photons by the sample with the unlabeled CIBN–CAAX component of the optogenetic system. The surface fluorescence of cells transfected with CIBN–CAAX alone was assessed at baseline and subsequent to illumination with blue light. The time-course of the change in fluorescence intensity (Fig. 3*B*) showed a small but statistically significant deviation from the baseline following activation of the “blue” laser. Because of the absence of a fluorophore in this experiment, the increase in signal is indicative of background noise and backscatter within the sample. Hence, the mean fluorescence following the activation of the “blue” laser was subtracted from surface fluorescence values after blue light illumination in all TIRFM experiments with mCherry-CRY2-5Ptase_OCRL_ and mCherry-CRY2–mPKCε-CAT-HA (Fig. 2). Cells transfected with mCherry-CRY2–mPKCε-CAT-HA alone were also assessed in TIRFM experiments (Fig. 3, *D*–*F*). Optogenetic recruitment of the CRY2-tagged enzyme failed to occur, emphasizing the ability of CIBN–CAAX and CRY2 to work in tandem to make optogenetic recruitment of mCherry-CRY2–mPKCε-CAT-HA possible.

### mCherry-CRY2–mPKCε-CAT-HA is recruited to the plasma membrane by the specific PKCε activator, FR236924

**Figure 4:**
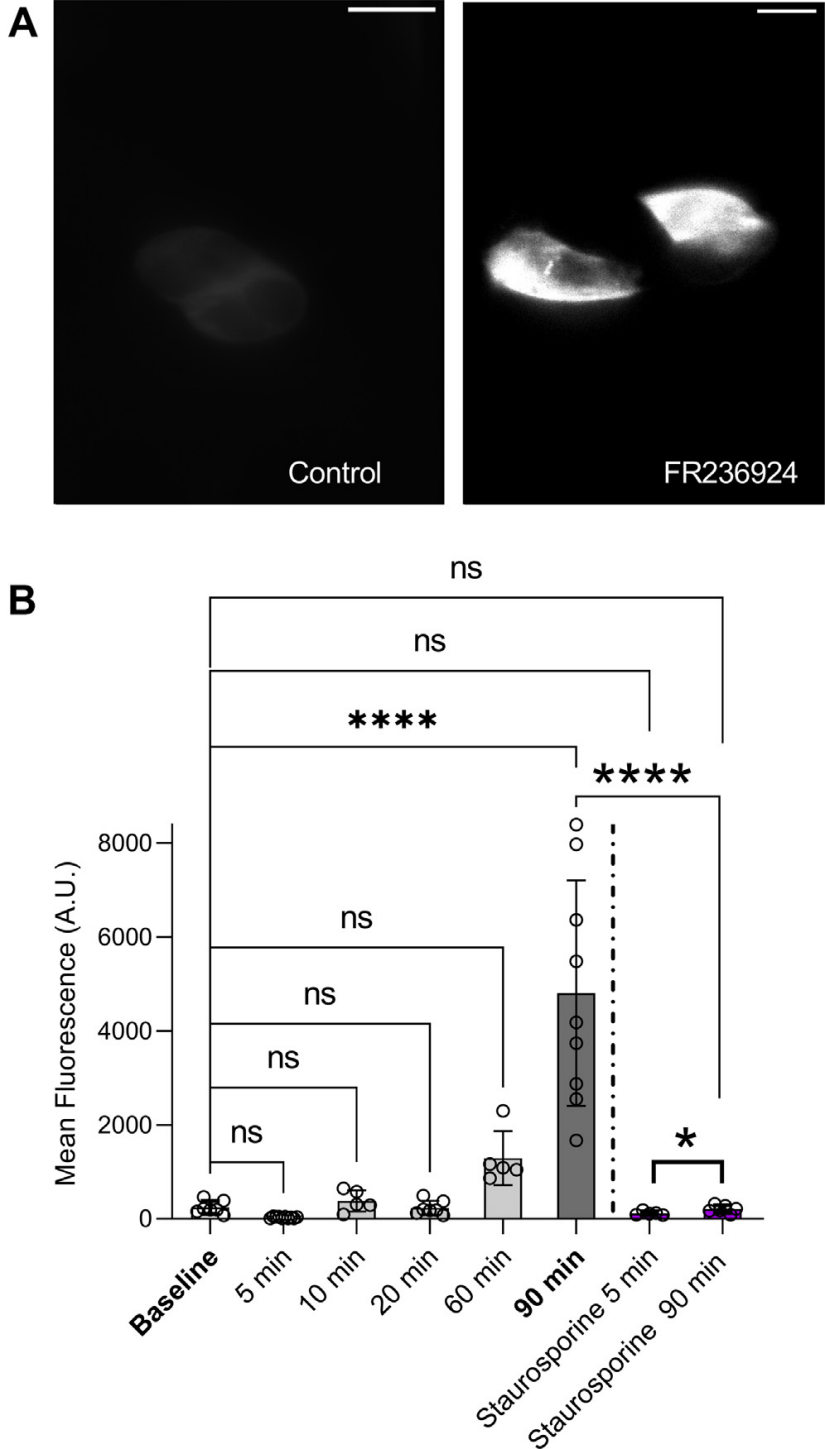
**The specific PKCε activator, FR236924, efficiently recruits mCherry-CRY2–mPKCε-CAT-HA to the cell membrane.***A*, representative TIRFM images of mCherry fluorescence at the cell surface show an increase in mCherry fluorescence after 90 min of treatment with 100 μM FR236924. *B*, summary data for mean cell surface fluorescence of mCherry following treatment with FR236924 at various time points, alone, or with 100 nM staurosporine. Data are mean fluorescence ± SD for 5 to 22 cells per experiment. One-way ANOVA with Sidak’s multiple comparison test, ∗∗∗∗*p* < 0.0001; two-way ANOVA for effect of staurosporine at 90 min with Tukey’s post hoc test. Scale bars represent 10 μM. CAT, catalytic domain; CRY2, cryptochrome-2; HA, hemagglutinin; TIRFM, total internal reflection fluorescence microscopy.

We used TIRFM to validate the activation of our construct by the specific PKCε activator, FR236924, as a positive control (11). Human embryonic kidney 293T (HEK-293T) cells were transfected with 0.75 μg of mCherry-CRY2–mPKCε-CAT-HA 24 h prior to analysis. The fluorescence of mCherry was tracked as a marker for expression of mCherry-CRY2–mPKCε-CAT-HA. The cell surface was brought into focus after which a 561 nm laser was used to excite mCherry fluorescence. Cells were incubated with 100 μM FR236924. Figure 4*B* shows a highly significant recruitment of mCherry-CRY2–mPKCε-CAT-HA to the cell membrane by FR236924 after 90 min of incubation. This surface localization of mCherry-CRY2–mPKCε-CAT-HA by FR2326924 was blocked by 100 nM staurosporine applied throughout the duration of FR236924 treatment.

### mCherry-CRY2–PKCε-CAT-HA phosphorylates the concatenated GIRK1/4 channel

**Figure 5:**
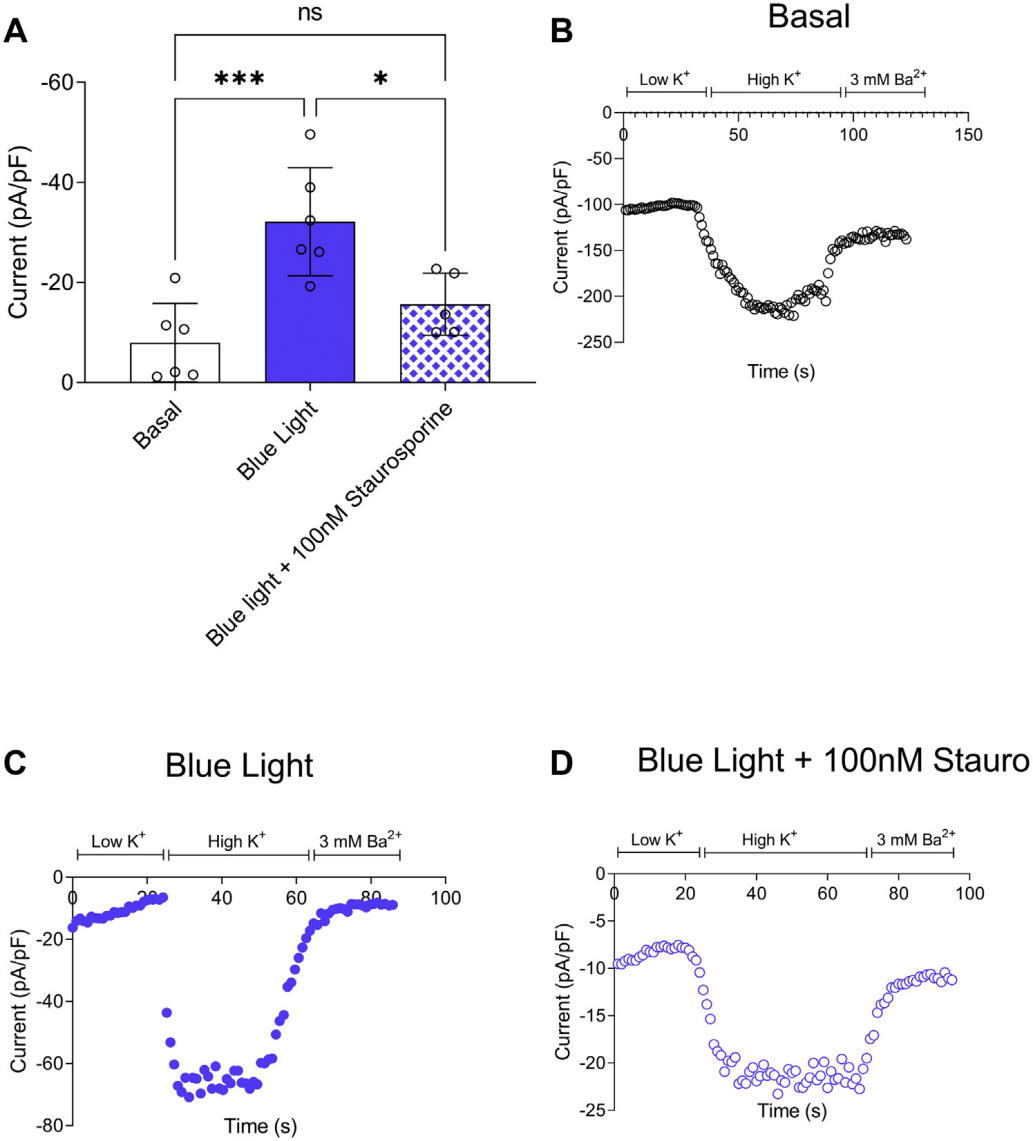
**mCherry-CRY2–mPKCε-CAT-HA activates GIRK1/4 concatemer activity in HEK-293T cells**. GIRK1/4 concatemers were expressed in HEK-293T cells with mCherry-CRY2–mPKCε-CAT-HA and CIBN-GFP-CAAX. Basal GIRK1/4 activity was evaluated in response to high K^+^ solution. Cells were illuminated with *blue light* (∼460 nm) for 40 min prior to electrophysiology experiments. *A*, GIRK1/4 activity is enhanced upon activation of mCherry-CRY2–mPKCε-CAT by 460λ. *B*, representative trace of GIRK1/4 activity before and (*C*) after illumination with *blue light* (460λ) (*D*) in the presence of 100 nM staurosporine. Data are Ba^2+^ subtracted, whole-cell currents expressed as mean ± SD (n = 5–6 cells per group). Negative currents indicate inward flow of positively charged ions; ∗∗∗*p* < 0.0005, ∗*p* < 0.05 calculated using ordinary one-way ANOVA. CAT, catalytic domain; CIBN, N-terminal region of the CRY2-binding domain of CIB1; CRY2, cryptochrome 2; GIRK1/4, G protein–gated inwardly rectifying K+ channel 1 and 4; HA, hemagglutinin; HEK-293T, human embryonic kidney 293T cell.

To provide functional evidence of the mCherry-CRY2–mPKCε-CAT-HA–mediated phosphorylation of specific membrane proteins, we assessed mCherry-CRY2–mPKCε-CAT-HA on the activity of an ion channel protein using whole-cell patch-clamp experiments. PKCε is reported to phosphorylate atrial GIRK channels in primary cells and enhance their activity (7). The phosphorylation of GIRK1/4 *via* kinase activity has been demonstrated to elevate channel-mediated potassium currents (7), but the precise phosphorylation site(s) remain(s) to be identified. To this end, we coexpressed mCherry-CRY2–mPKCε-CAT-HA with concatenated GIRK1/4, which is the predominant GIRK channel heterotetrameric complex expressed in atrial myocytes. GIRK1/4 currents following optogenetic activation of mCherry-CRY2–mPKCε-CAT-HA in real time during whole-cell patch-clamp experiments did not show any effect through the duration of the patch-clamp recording (data not shown), presumably because of a long timescale of the phosphorylation event. HEK-293T cells transfected with CIBN-GFP-CAAX, GIRK1/4 concatemer, and mCherry-CRY2–mPKCε-CAT-HA were illuminated with blue light for 40 min before patch-clamp electrophysiology experiments. We observed that cells illuminated with blue light demonstrated greater channel activity (mean current density −26.5 ± 15.2 pA/pF, n = 6; Fig. 5, *A* and *C*) compared with cells that were not (mean current density −7.9 ± 7.9 pA/pF, n = 6; Fig. 5, *A* and *B*). The enhancement of GIRK1/4 activity was abolished in the presence of 100 nM staurosporine. These data suggest that recruitment of PKCε induced by blue light results in GIRK1/4 channel phosphorylation causing elevation of GIRK1/4 activity.

## Discussion

We have shown that optogenetic recruitment of PKCε is not only a viable approach to study membrane proteins but also shows comparable results to using specific activator compounds (FR236924). In contrast, our optogenetic tool rapidly recruits the PKC isozyme to the plasma membrane.

We have had a good deal of success with constructs described by Idevall-Hagren *et al*. (5) for hydrolysis of phosphatidylinositol (4, 5) bisphosphate in patch-clamp electrophysiology experiments (9, 12). The data described in this article provide further evidence in favor of this optogenetic system and its wide applicability. The dimerization between the CRY2 construct and the membrane-anchored CIBN is rapid, as is evident by our TIRFM data; the functionality of this system appears to be limited by the enzyme to which CRY2 is linked. The phosphatase construct—mCherry-CRY2-5Ptase_OCRL_—produces phosphatidylinositol 4,5 bisphosphate dephosphorylation, observed in the form of macroscopic current changes, within seconds (9, 12). Because of the dynamic equilibrium of kinases and phosphatases that persists to maintain cellular homeostasis, it is possible that the longer time required in observing protein regulation than phosphoinositide dephosphorylation effects after blue light exposure is due to a gradual shift in equilibrium toward PKCε-mediated phosphorylation events.

With the ubiquitous application of the field of optogenetics to *in vivo* and *in vitro* work, alike, the opportunity to apply this strategy to study multiple PKC isozymes widens the scope of studies that are testing for specific phosphorylation through membrane targeting of the catalytic domain of each PKC isozyme.

## Experimental procedures

### Materials

The selective PKCε activator, FR236924, and staurosporine were purchased from Fisher Scientific. mPKCε-CAT-HA was a gift from Bernard Weinstein (Addgene; plasmid 211242).

### Constructing mCherry-CRY2–mPKCε-CAT-HA plasmid DNA

Using a construct developed by the De Camilli laboratory described in the study by Idevall-Hagren *et al.* (5), we replaced the inositol-5-phosphatase with the catalytic domain of murine PKCε to make mCherry-CRY2–mPKCε-CAT-HA. The partner construct containing the CIBN was engineered with a C-terminal CAAX box that targets it to the plasma membrane by prenylation. mCherry-CRY2-5Ptase_OCRL_ was digested with the restriction endonucleases PvuI and XmaI. The digestion products were then separated on an agarose gel, and the fragment corresponding to mCherry-CRY2 was excised from the agarose gel. Similarly, the HA-tagged catalytic subunit of mouse PKC in the vector, pXOOM, was digested with the restriction enzyme BamHI, after which the products were separated on an agarose gel to enable excision of the mPKCε-CAT-HA insert. The excised gel pieces were each dissolved in three gel volumes of QG buffer (5.5 M guanidine thiocyanate and 20 mM Tris–HCl [pH 6.6]). Once dissolved, one gel volume of isopropanol was added to enhance DNA yield. The solution was then applied to a QIAquick column and centrifuged for 1 min. The column was washed with PE buffer (10 mM Tris–HCl [pH 7.5] and 80% ethanol) and centrifuged before eluting with water. The purified mCherry-CRY2 fragment was then blunted with DNA polymerase I (Klenow) to remove 3′ overhangs of PvuI by 3′ —> 5′ exonuclease activity of Klenow and fill-in 5′ overhangs of XmaI by 5′ —> 3′polymerase activity. Overhangs of 5′ mPKCε-CAT-HA fragment created by BamHI were blunted by Klenow. The blunt ends of mCherry-CRY2 (as a vector) and mPKCε-CAT-HA (as an insert) were ligated using T4 DNA ligase (New England Biolabs) following the manufacturer’s protocol.

### Cell culture and transfection

HEK-293T cells were cultured in Dulbecco's modified Eagle's medium (Hyclone) supplemented with 1% penicillin/streptomycin and 10% fetal bovine serum and maintained at 5% CO_2_ at 37 °C. Cells were seeded on glass coverslips in 35 mm culture dishes for TIRFM and electrophysiology experiments at least 1 day before transfection. Cells were transiently transfected in OptiMEM using polyethyleneimine (PEI) for 2 to 6 h at a ratio of 1 μg of DNA to 4 μl PEI. Cells were studied in whole-cell patch-clamp experiments 24 to 48 h after transfection. Transfections for phosphor-staining experiments were performed in 6 cm plates.

### TIRFM

Cells seeded on 15 mm #1.5 coverslips were transfected using PEI (1:4) with 0.75 μg each of mCherry-CRY2–mPKCε-CAT-HA and CIBN–CAAX or mCherry-CRY2-5Ptase_OCRL_ and CIBN–CAAX 48 h before analysis. Cells were studied in a solution comprised of (in millimolar) NaCl 130, KCl 4, MgCl_2_ 1.2, CaCl_2_ 2, and Hepes 10, and pH was adjusted to 7.4 with NaOH. Blue light activation was performed using a 445-nm laser, and mCherry was excited by a 561-nm laser (Coherent). The beams were conditioned for coherence with custom-built Keplerian beam expanders upstream of laser cleanup filters (445/10 nm and 561/10×). Laser lines were tuned to provide 10 mW of incident light on a micromirror positioned below a high numerical aperture apochromat objective (60×, 1.5 numerical aperture; Olympus) mounted on an RM21 microscope frame equipped with a piezo-driven nanopositioning stage (Mad City Labs, Inc). The emission of mitochondrial fission process protein 1 (mTFP1) and mCherry was isolated from the excitation beam by an exit micromirror and a ring diagram positioned below the micromirror assembly. mCherry was imaged through 620/60 nm bandpass filters using a back-illuminated sCMOS camera (Teledyne Photometrics) controlled by Micro-Manager freeware (University of California San Francisco). All filters and mirrors were from Chroma. Lenses, pinholes, and diaphragms were from ThorLabs. TetraSpeck beads (Thermo) were routinely imaged to map the sCMOS chip and calibrate the evanescent field depth to 100 nm. To assess stoichiometry, fluorophores were photobleached by continual excitation. Images were captured at 2 to 5 s intervals, as indicated, to prevent bleaching, and the data were saved as separate stacks. Baseline (preillumination) values were taken before blue light illumination, and fluorescence at the last time point of data collection after blue light illumination was taken for postillumination. Image analysis and background subtraction were performed with ImageJ (National Institutes of Health).

### Whole-cell patch clamp

Electrophysiology experiments were performed at room temperature. To demonstrate mCherry-CRY2–mPKCε-CAT-HA translocation, cells were illuminated with blue light for 40 min. For experiments with staurosporine, coverslips were incubated with 100 nM staurosporine during blue light treatment. Whole-cell currents were recorded using a Tecella amplifier with the WinWCP software (University of Strathclyde). Currents were filtered at 2 kHz and digitized at 10 kHz. Borosilicate glass electrodes were pulled using a vertical puller (Narishige). Electrodes had a resistance of 2 to 5 MΩ when filled with an intracellular buffer comprised of 140 mM KCl, 2 mM MgCl_2_, 1 mM EGTA, 5 mM Na_2_ATP, 0.1 mM Na_2_GTP, and 5 mM Hepes buffered to pH 7.4 using KOH. Cell capacitances ranged from 8 to 15 pF. Cells were cotransfected with CIBN-GFP-CAAX. GFP+ (green) cells were selected for analysis using a Nikon epifluorescence microscope. Cells were perfused directly using a multibarrel gravity-driven perfusion apparatus and were initially perfused with a physiological buffer containing 135 mM NaCl, 5 mM KCl, 1.2 mM MgCl_2_, 1.5 mM CaCl_2_, 8 mM glucose, and 10 mM Hepes (pH 7.4). After a Giga-Ω seal was established with the patch pipette, slight suction was applied to the cells to access the whole-cell mode. Cells were held at 0 mV, and currents were recorded using a repeating ramp protocol from −80 mV to +80 mV in the whole-cell mode. GIRK channel activity was measured at −80 mV after transitioning to a high K^+^ buffer comprised of 5 mM NaCl, 140 mM KCl, 1.2 mM MgCl_2_, 1.5 mM, CaCl_2_, 8 mM glucose, and 10 mM Hepes (pH 7.4). Currents were blocked using 5 mM Ba^2+^ prepared in high K^+^.

### Statistics

All error bars represent the SD. Statistical significance was assessed using Student’s *t* test or ordinary one-way or two-way ANOVA in GraphPad Prism (GraphPad Software, Inc). Statistical significance was set at *p* < 0.05.

## Data availability

The data used to support the findings of this study are included within the article.

## Conflict of interest

The authors declare that they have no conflicts of interest with the contents of this article.

## References

[1] Newton A.C. (2010). Protein kinase C: Poised to signal. Am. J. Physiol. Endocrinol. Metab..

[2] Kikkawa U. (2019). The story of PKC: A discovery marked by unexpected twists and turns. IUBMB Life.

[3] Hug H., Sarre T.F. (1993). Protein kinase C isoenzymes: Divergence in signal transduction?. Biochem. J..

[4] Newton A.C. (2018). Protein kinase C as a tumor suppressor. Semin. Cancer Biol.

[5] Idevall-Hagren O., Dickson E.J., Hille B., Toomre D.K., De Camilli P. (2012). Optogenetic control of phosphoinositide metabolism. Proc. Natl. Acad. Sci. U. S. A..

[6] Kanno T., Yaguchi T., Nagata T., Tanaka A., Nishizaki T. (2009). DCP-LA stimulates AMPA receptor exocytosis through CaMKII activation due to PP-1 inhibition. J. Cell Physiol..

[7] Makary S., Voigt N., Maguy A., Wakili R., Nishida K., Harada M., Dobrev D., Nattel S. (2011). Differential protein kinase C isoform regulation and increased constitutive activity of acetylcholine-regulated potassium channels in atrial remodeling. Circ. Res..

[8] Schleifenbaum A., Stier G., Gasch A., Sattler M., Schultz C. (2004). Genetically encoded FRET probe for PKC activity based on pleckstrin. J. Am. Chem. Soc..

[9] Xu Y., Cantwell L., Molosh A.I., Plant L.D., Gazgalis D., Fitz S.D., Dustrude E.T., Yang Y., Kawano T., Garai S. (2020). The small molecule GAT1508 activates brain-specific GIRK1/2 channel heteromers and facilitates conditioned fear extinction in rodents. J. Biol. Chem..

[10] Liu H., Yu X., Li K., Klejnot J., Yang H., Lisiero D., Lin C. (2008). Photoexcited CRY2 interacts with CIB1 to regulate transcription and floral initiation in arabidopsis. Science.

[11] Kanno T., Yamamoto H., Yaguchi T., Hi R., Mukasa T., Fujikawa H., Nagata T., Yamamoto S., Tanaka A., Nishizaki T. (2006). The linoleic acid derivative DCP-LA selectively activates PKC-ϵ, possibly binding to the phosphatidylserine binding site. J. Lipid Res..

[12] Ningoo M., Plant L.D., Greka A., Logothetis D.E. (2021). PIP2 regulation of TRPC5 channel activation and desensitization. J. Biol. Chem..

